# How people perceive malicious comments differently: factors influencing the perception of maliciousness in online news comments

**DOI:** 10.3389/fpsyg.2023.1221005

**Published:** 2023-08-22

**Authors:** Saerom Lee, Hyunmi Baek, Seongcheol Kim

**Affiliations:** ^1^School of Business Administration, Kyungpook National University, Daegu, Republic of Korea; ^2^School of Media and Communication, Korea University, Seoul, Republic of Korea

**Keywords:** perceived maliciousness, online news comments, malicious comments, individual factors, contextual factors

## Abstract

This study proposes a comprehensive model to investigate the factors that influence the perceived maliciousness of online news comments. The study specifically examines individual factors, including demographic characteristics (e.g., gender and age), personality traits (e.g., empathy and attitudes toward online news comments), and reading-related factors (e.g., the amount of news comment reading). Contextual factors such as issue involvement, perceived peer behavior, and the presence of malicious comments in news articles are also considered. The results suggest that most of the proposed variables have a significant impact on the perceived maliciousness of online news comments, except for morality and issue involvement. The findings have important theoretical implications for research on malicious online news comments and provide practical guidelines for online news platforms on how to reduce malicious comments by visualizing them alongside other news comments.

## 1. Introduction

People can read others' thoughts about particular issues on the news from online news comments. Online news comments can also monitor or check journalists and provide opportunities for democratic debate (Santana, 2011; Anderson et al., 2014; Weber, 2014). Behind these positive impacts, there are serious adverse impacts of online news comments such as malicious comments. Malicious comments are defined as “making rude or nasty comments to someone on the internet” (Ybarra and Mitchell, 2004, p. 1,310). Malicious online news comments deepen serious conflicts and hate cultures between generations and genders (Lewandowska-Tomaszczyk, 2017). During the pandemic period spanning from 2019 to mid-2022, there was a marked increase of over 20% in the incidence of malign comments, while hate speech directed toward particular ethnic groups or individuals has exhibited an escalating tendency toward extremity (BBC News, 2021). Furthermore, cyberbullying using malicious comments against certain people is causing severe social problems (Lee et al., 2011) that can lead to depression or suicide in targeted groups or individuals. To solve the problem of malicious online news comments, the online news platforms are also seeking various ways, such as automatically detecting malicious comments, abolishing the comment threads of a particular news section, or providing commenters’ commenting history information.

In academic areas, various research has consistently identified the problems and solutions of malicious online news comments (Coe et al., 2014; Freelon, 2015; Lee and McElroy, 2019). For example, previous research mainly focused on the impact of malicious comments (Borah, 2013; Anderson et al., 2014; Gervais, 2015; Mutz, 2015), the cause of malicious comments (Massaro and Stryker, 2012; Coe et al., 2014; Santana, 2014; Rowe, 2015), technical approach to reduce malicious comments (Badjatiya et al., 2017; Davidson et al., 2017; Risch and Krestel, 2020) and the contents of malicious comments (Herbst, 2010; Muddiman, 2013; Berry and Sobieraj, 2014).

Malicious online news comments are a recent cyber deviant behavior, with undefined definitions and boundaries. Previous research has focused on individual factors affecting deviant online comment behavior(Kim and Kim, 2018; Kenski et al., 2020). However, there is a lack of research on the impact of online news comments as contextual cues, particularly how malicious comments in the same article influence perceived maliciousness. Context factors, such as perceived peer behavior on malicious commenting, affect the processing of information about comments and perceived maliciousness (Lee et al., 2011). Additionally, the reader's degree of involvement in the article content affects cognitive ability in the process of acquiring comments and judgment of perceived maliciousness (Petty and Cacioppo, 1986). Therefore, this research proposes a comprehensive examination of the antecedents of perceived maliciousness of online news comments.

The purpose of this research are as follow: we revisit the previous research factors affecting individuals' perceived maliciousness of online news comments in order to find ways to reduce malicious comments more fundamentally. Specifically, this study combines the individual and contextual factors from previous research and suggests a comprehensive model with demographic characteristics, personality, comment reading-related factors (e.g. new comments reading amount), and contextual factors (e.g. issue involvement, presence of malicious comments in the comment sections, and perceived peer’ behavior on malicious commenting).

The organization of this article is as follows. In the next section, relevant prior literature is examined to establish a theoretical foundation. This is followed by a presentation of our research hypotheses. Next, the research design and analysis for the survey are described. Finally, this study concludes with a discussion of implications for research and practice, limitations, and future research extensions.

## 2. Literature review and hypotheses

### 2.1. Previous research on malicious online news comments

Online news comments are important as a way for expressing individual opinions, but the negative social influence of malicious comments cannot be ignored. Malicious comments are continuously mentioned as social problems due to negative public opinion formation, conflict, suicide, etc. (Lee et al., 2011). Malicious online news comments are relatively recent cyber deviant behaviors. Thus, there is a lack of well-defined definitions and boundaries of malicious online news comments. Additionally, as the pre-step of writing malicious comments, it is necessary to understand what kinds of factors affect the perceived maliciousness in individuals’ minds. We define “perceived maliciousness” as to how people perceive rude or nasty online news comments to someone sensitively. Specifically, Orenga et al. (2000) indicate that people use malicious language more prevalently in computer-mediated communication environments, unlike face-to-face environments. We systematically review the streams of research on malicious online news comments such as impacts of malicious comments, motivations, antecedents on the degree of malicious comments, the strategy of reducing malicious comment, and characteristics of malicious commenters.

First, previous studies have examined how malicious comments affect emotions or opinions. Hwang et al. (2018) stated that exposure to malicious discussion increases the negative emotion of the opponent, affecting disagreement about the discussion. Kwon and Cho (2017) conducted an analysis of user comments obtained from 26 newspaper websites, and showed that swearing plays a positive role in bringing user attention to comments. There are previous studies on the motivation for writing malicious comments such as anonymity (Moore et al., 2012; Kim et al., 2020), morality (Hwang et al., 2016), assertiveness (Alonzo and Aiken, 2004), low self-control (Lee and Kim, 2015). Because of online anonymity and lack of real-world norms, people tend to ignore or behave rudely online (Kiesler et al., 1984, 1985). Additionally, Lee (2009) described the motivation of writing malicious comments for fun, curiosity, engaging and showing off, sympathizing with his group, expressing exclusive emotions, disliking certain people, and recognizing them as parts of the discussion process.

Second, there are studies that deal with the factors that affect the degree of malicious comments (Coe et al., 2014; Su et al., 2018; Wang and Silva, 2018). Coe et al. (2014) analyzed the patterns and determinants of malicious comments in more than 6,400 online news comments published in 300 news articles. The results indicate that contextual factors such as topics of the articles and sources quoted within articles can affect malicious comments. In addition, Su et al. (2018) showed that the degree of malicious comments can vary depending on news outlets. Wang and Silva (2018) also found that the degree of malicious comments is influenced by topics of articles. Most previous research descriptively has focused on the certain news section where malicious comments are frequently written. For example, Coe et al. (2014) indicated that malicious comments posit greater in hard news sections such as politics, law, and foreign affairs.

Third, other stream of research on malicious comments focus on the way to prevent or reduce the malicious comments. There are many existing studies that show that people are more likely to commit crimes in a computer-based communication environment because their anonymity is guaranteed (Coffey and Woolworth, 2004). In the same vein, research about malicious comments has continued to show that the likelihood of writing malicious comments increases in anonymity setting (Moore et al., 2012; Cho and Kwon, 2015; Kim et al., 2020). In order to solve this problem, portal sites have made efforts to reduce malicious comments by changing the platform policy such as introducing the internet real-name system or linking their social network accounts (Cho and Kwon, 2015). In addition, news platform operators try to adopt news comment ordering systems to reduce malicious comments. For example, Baek et al. (2020) analyzed the impacts of changing comments ordering system on malicious comments in Naver, the dominant news platform in Korea. Technically, portal sites are trying to improve the platform from a long-term perspective by introducing a clean bot to automatically delete malicious comments or to manage the history of commenters by disclosing their past comment writing history.

Finally, studies have been conducted on the characteristics of malicious commenters. Lee and Park (2019) empirically analyzed the demographic characteristics of malicious commenters. According to their research, people in their 40s and 50s use a lot of profanities in comments. Baek et al. (2022) also tried to identify the characteristics of malicious commenters using datasets from the Naver news portal. They found that males and older people tend to write more malicious comments, and the news commenters who leave and delete more comments, and leave longer comments tend to be malicious commenters. Commenters who wrote malicious comments have shown that they tend not to use evidence for their claims compared to commenters who do not.

### 2.2. Perceived maliciousness of online news comments

Writing malicious online news comments are a relatively recent emerging deviant behavior in the internet environment. Malicious comments do not cause direct injury but cause indirect damage, so people who write malicious comments do not perceive exactly what they are doing. Crimes such as dating violence also have characteristics that the perpetrator or society cannot easily perceive it as violence even though the victim's suffering occurs continuously and repeatedly in everyday life (Lee, 2018). Therefore, depending on the individual's perceptions, there are possibilities that the same behavior is considered sexual harassment or not (Gutek et al., 1983; Kim et al., 2001). In case of online news comments, although people read the same malicious online news comments, they can accept the comments differently according to their sensitivities and perceptions toward malicious comments. Moreover, the extent of the damage is not accurately recognized because the damage happens under a non-face-to-face relationship between the malicious commenters and those who are affected. Therefore, it is difficult to recognize whether the comments written by the perpetrator are malicious comments or not. Thus, the purpose of this research focuses on the fundamental approach to reveal the antecedents that cause the differences of perceived maliciousness of online news comments. For example, Kenski et al. (2020) conducted a study of how certain message types or individual characteristics affect the perceptions of maliciousness in online discourse. They adopted twelve types of online news comments from the survey of Coe et al. (2014) to evaluate the perception of the relative maliciousness for each comment. The results indicate that different types of online news comments lead to different responses from readers. Concretely, online news comments with name-calling and vulgarity are rate high as perceived malicious comments than other types of comments. Kim and Kim (2018) also examine the impacts of others’ malicious comments and observes the response of readers. The research adopts the social identity group and explains that people response differently to malicious comment if the comments is generated from out-group members who have an opposite political opinion. However, there are a lack of research that precisely targets to examine the antecedents of readers’ perceived maliciousness of online news comments. In this vein, we suggest the research question about what makes differences in perceiving maliciousness of online news comments. How do perceived maliciousness of online news comments differ based on individual characteristics, and what are the factors that influence these differences? More specifically, what can be the individual factors and contextual factors that affect the perceived maliciousness of online news comments?

### 2.3. Individual factors

In this research, we consider demographic information such as gender and age to predict the perceived maliciousness of online news comments. In case of gender, the researchers found that men write more malicious comments than women (Moss-Racusin et al., 2015; Baek et al., 2022). Kenski et al. (2020) considered demographic information as antecedents of perception of maliciousness on online news comments. According to the analysis, women perceive maliciousness more sensitively than men. Furthermore, psychologist Hyde (2005) conducted a meta-analysis of the differences in personality traits between men and women. The results show that the aggression of men scored higher than women, which means men is more aggressive than women. Based on the Hyde’s results, we can assume that men can be more aggressively response to the news articles with malicious comments. In the case of age, we adopt social learning theory (Akers, 2011) as a theoretical lens to interpret the impacts of becoming old. According to social learning theory, individual criminal behavior is formed through the learning of social culture. The older you get, the more opportunities you have to learn. Then, there are more possibilities that older people might commit criminal acts. Therefore, it is possible that older people has less sensitive toward swearing or rude comments. Baek et al. (2022) empirically showed that older people tend to write more malicious comments. Thus, we suggest hypotheses as below:

*H1-1*: Females will more sensitively perceive the maliciousness of online news comments than males.

*H1-2*: Older people will less sensitively perceive the maliciousness of online news comments than younger people.

Morality refer to “prohibitions and prescriptions, at the core of which are norms prohibiting harm to relevant others” (Maibom, 2009, p. 483). Morality is known to strongly influence controlling deviant behavior in various contexts (Moores and Chang, 2006; Hu et al., 2011). It makes individuals feel shame and guilt when they commit a deviant behavior (Wikström et al., 2012; Xu et al., 2016). In other words, people with high morality show strong disapproval of a deviant behavior (Lewis, 1971; Lowry et al., 2016). Aquino et al. (2009) stated that people with strong internalization of their moral identity behave more cooperatively and pro-social compared to those who do not. Individuals who have learned that malicious comments are considered immoral will hesitate to write malicious comments (Kim and Kim, 2018). Kim et al. (2020) contented that situational morality can control malicious comment writing behaviors because the negative feeling led by high morality make people feel guilt and shame about their deviant behavior. For example, individuals with high moral beliefs has less attempt to commit software privacy (Siponen et al., 2012), less likely to expose other’s sensitive information online (Xu et al., 2016). In this regard, people with high morality will respond more sensitively to the perceived maliciousness of online news comments.

*H2-1*: Morality will positively influence the perceived maliciousness of online news comments.

Empathy is basically other-oriented concerns or motivations which head to increase the others’ well-being or welfare (Batson and Shaw, 1991). Empathy is the basis for prosocial and altruistic behavior by sharing emotional experiences and understanding other people's emotions. Therefore, empathy is one of the main factors that cause cognitive distortions in the situation of committing crimes (Song et al., 2008). For example, Aos et al. (2001) contended that low empathy increases the likelihood of committing a crime by causing the perpetrator's cognitive distortion of sexual assault crimes. Kenski et al. (2020) investigated that people with high levels of agreeableness, which is based on empathy, also showed a high score in the perception of malicious speech. Empathy will lead to an understanding of the damage that a malicious comment target can receive. Thus, we suggest hypothesis as below:

*H2-2*: Empathy will positively influence the perceived maliciousness of online news comments.

Attitude refer to a psychological tendency that is expressed by evaluating a particular entity with some degree of favor or disfavor (Eagly and Chaiken, 1993. p. 1). Jung and Lee (2009) said that those who have a positive attitude toward news comments are relatively positive about the quality of the comments. In this study, it is assumed that if the attitude toward the comment itself is positive and feels the need, it will be relatively less sensitive in perceiving whether a particular online news comment contains maliciousness or not. In this vein, if people feel that news comments are necessary, you may judge the contents of the comments more positively and your perceived maliciousness of online news comments may be less sensitive. Therefore, we develop hypotheses as below:

*H3:* Positive attitude toward online comments will negatively influence the perceived maliciousness of online news comments.

Existing studies have shown that the more a specific media is used, the higher the tendency to believe that the media provides credible information (Flanagin and Metzger, 2000; Schweiger, 2000). Jung and Lee (2009) contented the amount of comment reading could have a positive effect on the evaluation of the quality of comments. Thus, we can assume that the heavy comments readers can lead to the high the credibility of the comments, which may increase the tendency to give a positive evaluation when evaluating comments. Additionally, to consider the process of automatic behavior formation, positive rewards of behavior will be stronger the behaviors (Chen, 2017). Then, we can assume that people who have comment reading behavior might gradually receive positive rewards and satisfaction from the comment reading such as get the helpful information form the comments or supportive opinion of others. Therefore, for heavy news comment readers, there are higher opportunities to read credible comments in daily life. Thus, heavy news comment readers perceive the maliciousness of comments relatively sensitively. Therefore, we suggest hypothesis as below:

*H4*: Heavy news comment readers will more sensitively perceive maliciousness of online news comments than light news comment readers.

### 2.4. Contextual factors

In this study, we try to verify how contextual factors affect the perceived maliciousness of online news comments. Specifically, contextual factors to which users may be exposed when reading news articles include certain news issue and other commenters’ comments about the news article. In addition, we also include environmental factors perceived by individuals such as peers’ behavior in the research model.

Issue involvement refers to “the extent to which the attitudinal issue under consideration of personal importance” (Petty and Cacioppo, 1979). With high issue involvement, people pay much attention to the information and arouse interest (Lee and Kim, 2016). In this vein, if news readers are exposed to comments on articles with high issue involvement, it is unlikely that opinions will change due to comments because you already had individual opinions on the issue (Lee and Lee, 2008; Lee and Jang, 2009). On the other hand, low issue involvement was likely to change opinions by comments because there were no opinions of individuals already formed. In this study, we assume that high level of issue involvement was interpreted as the extent to which individuals previously formed opinions on the issue. Therefore, we suggest hypothesis as below:

*H5*: Issue involvement will negatively influence the perceived maliciousness of online news comments.

Previous research analyzed whether online news comments affect the perception of articles (Lee and Jang, 2009). As a result of the experiment, the researchers confirmed that the experiment subjects judge the news content more negatively when they read negative online news comments written by many people than when they do not read the online news comments. In addition, Yang (2008) also stated that the tone of comments has a greater influence on people's opinions than the tone of articles. As such, online news comments can be a contextual factor that can influence an individual to form an impression on a specific subject. However, there are lack of research that examine the impacts of online news comments on other online news comments. To consider the Aker’s social learning theory (SLT), which emphasizes the others’ behavior when we learn the boundary of behavior (Khansa et al., 2017), a lot of malicious comments in online news sections make people insensitive to certain malicious comments when they judge them. In this study, we divide two different survey group to investigate the impacts of other online news comments in the same news article. First, respondents in the first group read many malicious comments when they read the targeted malicious comment on the same news article. Second, we provide no malicious comments except the targeted malicious comments to respondents of the second group. We assume that when there are no malicious comments, people are well aware of the severity of malicious comments. On the other hand, when there are many malicious comments on the same news article, people seldomly recognize that the targeted comment is malicious. Thus, we hypothesize as below:

*H6*: The more other malicious comments on the same news article, people will perceive the maliciousness of online news comments less sensitively.

Peer influence refers to a set of process whereby one people affected by other’s behaviors. In this research, we adopt concept of peer influence as perceived peer’ behavior on malicious commenting relating to how peoples perceived the others’ malicious commenting behaviors. Among the components described in Akers' SLT, the differential association explains that peer influence affects deviant behaviors (Khansa et al., 2017). The more opportunities for differential associations on criminal behavior, the more opportunities for learning them. If a person responds positively to a particular criminal act, he or she may strengthen the value of the norm for that crime. Based on social learning theory, Lowry et al. (2016) also explained that peer influence can affect online deviant behavior. If people are aware that their peers write malicious online comments frequently, they might be insensitive to the malicious comments. Therefore, we establish the following hypothesis.

*H7*: Perceived peer’ behavior on malicious commenting will negatively influence the perceived maliciousness of online news comments.

**Figure 1:**
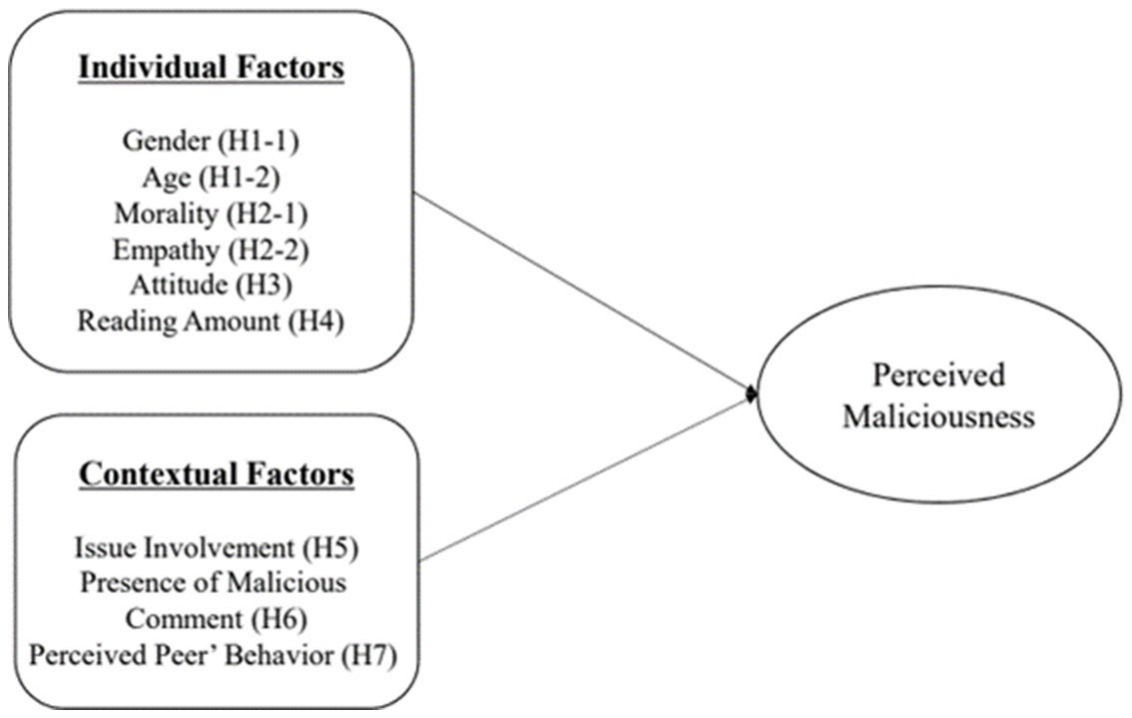
Research model.

In Figure 1. we suggest our research model based on the hypotheses.

## 3. Research methodology

### 3.1. Data collection

An online survey was conducted with 1,000 people from September 16 to September 21, 2020 through the survey company, Macromill Embrain. In the current study, certain sections of online news sites in Korea have implemented functional restrictions, such as blocking the comment feature, to prevent the occurrence of malicious comments. However, since the focus of this research is to examine individuals' perceptions of malicious comments they encounter, it is unrelated to these functional limitations. Instead, the study aimed to investigate and classify malicious comments on sports news based on a scenario. Therefore, the scenario used in this research was developed independently and is not directly related to the comment feature on online news sites in Korea. By conducting a survey considering age and gender, out of 1,000 respondents, 500 males and females each, and 200 respondents each from their 20s to 60s. The Table 1 shows the distribution of gender, age, education level, and household income of respondents. The table also shows the distribution of each respondent based on the answer to the question about whether you have read or written comments in the past week.

**Table 1:** The profile of the respondents.

Characteristics		Total No	People who read news comments		People who write news comments	
			No	Percentage	No	Percentage
All		1000	783	78.30%	144	14.40%
Gender	Male	500	389	77.80%	87	17.40%
	Female	500	394	78.80%	57	11.40%
Age	20s	200	174	87.00%	27	13.50%
	30s	200	168	84.00%	29	14.50%
	40s	200	146	73.00%	24	12.00%
	50s	200	155	77.50%	35	17.50%
	60s	200	140	70.00%	29	14.50%
Education	Below high school	5	3	60.00%	0	0.00%
	High school or equivalent	145	98	67.59%	26	17.93%
	Junior college or equivalent degree	146	109	74.66%	18	12.33%
	Bachelor’s or equivalent degree	597	490	82.08%	87	14.57%
	Postgraduate degree	107	83	77.57%	13	12.15%
Household income	1 million Korean won or Less	69	46	66.67%	11	15.94%
	1 – 3 million Korean won	257	199	77.43%	39	15.18%
	3 – 6 million Korean won	444	342	77.03%	55	12.39%
	More than 6 million Korean won	230	196	85.22%	39	16.96%

^*^The percent value is derived based on the total number of each row.

The percentage of people who read comments was 783 out of a total of 1000 people (78.3%), whereas the percentage of people who wrote comments in the last week was only 14.4%. Regarding the behavior of reading comments, there was almost no difference between men and women (male: 77.8%, female: 78.8%). In terms of age, it was found that in their 20s (87.0%) and in their 30s (84.0%), they read the most comments. In addition, in the case of university graduation and enrollment, the rate of reading comments was the highest at 82.1%, and the rate of reading comments from high income earners (over 6 million won) was 85.22%.

17.4% of men write comments, while 11.4% of women write comments, showing differences between men and women. In the order of 50s (17.5%), 60s (14.5%) and 30s (14.5%), they were found to be active in commenting. These findings are consistent with Ziegele et al. (2013) showing that male and old people participate in commenting actively through a survey conducted in Germany. Those who graduated from high school (17.9%) are the most active in writing comments, and those with high income (more than 6 million won, 17.0%) or low income (less than 1 million won, 16%) are the most active in writing comments.

### 3.2. Questionnaire and measures

This research model has nine independent variables and one dependent variable. Among the variables, except age, genders, reading amount and presence of malicious comments, other six constructs adopt multi-items for validity. Each item is gauged by a seven-point Likert scale. The measures of the constructs are adopted from literature except the perceived maliciousness. Table 2 shows the measures of the research constructs.

**Table 2:** Questionnaire items.

Construct	Item	Item description	Source
Attitude	AC1	Online news comments are helpful in establishing my opinion on the content of the news article.	Jung and Lee (2009)
	AC2	Online news comments are helpful for getting new information.	
	AC3	Online news comments are helpful in determining if my opinion is correct.	
	AC4	Online news comments provide evidence for setting my attitude toward a particular issue.	
Morality	M1	Most of my decisions are taking into account others.	Penner et al. (1995)
	M2	I choose actions that can help others as much as I can.	
Empathy	E1	I often feel anxious about someone who is less fortunate than me.	Penner et al. (1995)
	E2	I sometimes try to understand them better, imagining from the point of view of my friends.	
Perceived peer’ behavior on malicious commenting	PI1	I believe most people write malicious comments from time to time.	Khansa et al. (2017)
	PI2	I think people around me write malicious comments.	
Issue Involvement	II1	The issue covered in the news article is important to me.	Yang (2008)
	II2	The issue addressed in the news article is of personal interest.	
Perceived maliciousness	PMC1	The comment is considered a malicious comment.	
	PMC2	The comment contains abusive language and slander about Choi Dong-soo.	

### 3.3. Result of hypotheses test

This study adopts PLS–SEM to analyze the collected data. In the case of PLS, a prediction-oriented estimation method is used because the goal is to maximize the predictive power of the estimation coefficient while minimizing the residual generated in the model estimation process (Vinzi et al., 2010). In this study, we aim to explore an exploratory phenomenon, and in PLS-SEM, the focus is more on validating the path coefficients between variables rather than improving the fit of the research model within existing theories. Therefore, we have chosen the more suitable approach of PLS-SEM, considering the large number of items for each variable. We have employed a methodology that incorporates statistical considerations for both the measurement model and the path model. Therefore, we apply the PLS-SEM as it was suitable for exploratory research using SmartPLS 3.0. The analysis contains two steps; the first step includes evaluating the measurement model, and the second step involves an assessment of the structural model.

#### 3.3.1. Measurement model

The questionnaire asked each respondent to provide demographic data including gender, age, income, education. Respondents also responded to the behavior of news reading, reading and writing comments, attitudes on comments, and personal traits. After this, participants read a news article in the sports section and 10 comments on the comments thread under the news article (See Appendix A). Respondents were divided into group A and group B according to the rate of malicious comments. In this study, a questionnaire was conducted by selecting sports articles. Although the problem of malicious online news comments in the political section is serious, it was judged that the pre-existed attitude toward the topic of the article could have a great influence on the perceived maliciousness in online news comment. Therefore, the topic was selected as a spot article that can control the prior attitude. Group A (500 people) read 10 comments with a low rate of malicious comments, and Group B (500 people) read 10 comments with a high rate of malicious comments. Both groups responded to a questionnaire about whether the target comment "a psychopath-like guy… trying to get rid of the head of a guy he doesn't like" are perceived malicious online news comments. Each question was measured on a 7-point Likert scale (Table 2).

**Table 3:** Cross loading.

	Morality	Empathy	Attitude	Issue involvement	Peer’ behavior	Perceived maliciousness
M1	**0.680**	0.363	0.165	0.094	−0.039	0.064
M2	**0.967**	0.671	0.122	0.065	−0.141	0.184
E1	0.594	**0.912**	0.058	0.003	−0.157	0.228
E2	0.592	**0.876**	0.146	0.095	−0.085	0.195
AC1	0.132	0.104	**0.761**	0.166	0.189	−0.013
AC2	0.189	0.157	**0.802**	0.160	0.187	−0.028
AC3	0.130	0.120	**0.894**	0.208	0.218	−0.052
AC4	0.119	0.068	**0.952**	0.198	0.255	−0.090
II1	0.064	0.038	0.240	**0.954**	0.183	−0.075
II2	0.091	0.059	0.175	**0.963**	0.160	−0.083
PI1	−0.079	−0.054	0.176	0.099	**0.820**	−0.092
PI2	−0.136	−0.158	0.264	0.192	**0.972**	−0.224
PMC1	0.154	0.224	−0.076	−0.074	−0.212	**0.971**
PMC2	0.179	0.237	−0.064	−0.087	−0.177	**0.971**

Loadings (in bold) and cross-loadings of the model constructs and their indicators.

Beyond considering reflective indicators, the process of assessing discriminant validity includes the examination of both loadings and cross-loadings. Validity is confirmed when the loading factor of an indicator for its intended construct is higher than that for other constructs, and when cross-loading values surpass the threshold of 0.5. This information, along with the loadings (emphasized) and cross-loadings of the model’s constructs and indicators, is presented in Table 3.

As Table 4 shows, the discriminant validity of the scales is assessed by comparing the square roots of the AVEs with the correlations among the ten constructs. The square roots of AVEs for each construct are greater than their correlations with any other construct, meaning that the constructs are empirically distinct.

**Table 4:** Analysis of discriminant validity.

Construct	Correlation and square roots of AVE									
	Perceived maliciousness	Gender	Age	Morality	Empathy	Attitude	Issue involvement	Reading amount	Perceived peer’ behavior on malicious commenting	Presence of malicious comments
Perceived maliciousness	0.971									
Gender	0.150*	n/a								
Age	−0.066*	−0.005	n/a							
Morality	0.087**	−0.044	0.053	0.836						
Empathy	0.237**	0.099**	0.124**	0.524**	00.894					
Attitude	−0.052	−0.024	−0.044	0.215**	0.131**	00.856				
Issue involvement	−0.083**	−0.284**	0.012	0.116**	0.054	0.210**	0.959			
Reading amount	0.179**	0.015	−0.168**	0.133**	0.159**	0.285**	0.099**	n/a		
Perceived peer’ behavior on malicious commenting	−0.173**	−0.143**	−0.013	−0.044	−0.114**	0.228**	0.054	0.090**	0.899	
Presence of malicious comment	−0.027	0.000	−0.004	0.015	−0.060	0.013	0.014	−0.065*	−0.017	n/a

Square root of AVEs on the diagonal; *p < .05, **p < .01, ***p < .001.

#### 3.3.2. Common method variance and multicollinearity

Common method variance. This study tests the common method variance analysis of Harman’s single-factor test (Podsakoff et al., 2003), and follows the suggests of Delerue and Lejeune (2010) regarding the total variance explained for one common factor, meaning below the cut-off point of 50 percent. The result accounts for 21.377 percent, which confirms that the common method bias in our data set should be acceptable. Based on the Kock (2015), full collinearity variance inflation factor (VIF) can also be used for common method bias testing. Since all the VIFs are lower than 1.5 meaning that the common method bias is not serious in this research.

Multicollinearity. The multicollinearity problem evaluates VIF. All constructs have VIF statistics examination to assess the multicollinearity problem. As the Table 5 shows, all the VIFs are lower than 1.5 indicating that the multicollinearity problem is not detected. According to Kline (2005), the results of this study meet the requirements (See Table 6).

**Table 5:** Construct reliability.

Construct	Item	Factor loading	VIF	Cronbach’s alpha	Composite reliability	AVE	Average	SD
Attitude	AC1	0.761	2.356	0.896	0.917	0.732	3.97	1.27
	AC2	0.802	2.391					
	AC3	0.894	2.801					
	AC4	0.952	2.722					
Morality	M1	0.680	1.285	0.640	0.818	0.699	4.69	0.97
	
	M2	0.967						
Empathy	E1	0.912	1.564	0.750	0.888	0.799	5.07	0.93
	E2	0.876						
Perceived peer’ behavior on malicious commenting	PI1	0.820	1.777	0.796	0.893	0.808	3.34	1.28
	PI2	0.972						
Issue involvement	II1	0.954	3.367	0.912	0.958	0.919	3.54	1.50
	II2	0.963						
Perceived maliciousness	PMC1	0.971	4.641	0.939	0.971	0.943	5.99	1.06
	
	PMC2	0.971						

**Table 6:** Results of multicollinearity testing.

Model 1	Unstandardized coefficients		Standardized coefficients			Collinearity statistics	
	B	SE	*β*	*t*	Sig.	Tolerance	VIF
Constant	5.208	0.284		18.321	0.000		
Attitude	−0.075	0.028	−0.089	−2.707	0.007	0.820	1.220
Morality	−0.029	0.039	−0.026	−0.742	0.458	0.690	1.448
Empathy	0.255	0.041	0.222	6.150	0.000	0.678	1.476
Perceived peer malicious commenting behavior	−0.105	0.026	−0.125	−4.012	0.000	0.900	1.111
Issue involvement	−0.031	0.023	−0.043	−1.362	0.173	0.864	1.157
Reading amount	0.079	0.014	0.175	5.504	0.000	0.866	1.155
Presence of malicious comments	−0.005	0.064	−0.002	−0.073	0.942	0.988	1.012
Age	−0.005	0.064	−0.002	−0.073	0.942	0.988	1.012
Gender	0.196	0.067	0.092	2.916	0.004	0.888	1.126

Dependent variable: perceived maliciousness.

In this study, we treated gender and age as key individual characteristic variables to be tested in our hypotheses not control variables. Particularly, with regard to malicious comments, there is a recurring pattern where specific age groups or genders are more prone to engage in such behavior. Therefore, in studies investigating the perception of malicious comments, it is important to examine the influence of personal demographic characteristics, such as gender and age, on the perception of malicious comments.

#### 3.3.3. Structural model

To assess the structural model, using bootstrapping procedure (3,000 re-samples) determines the significance of the paths within the structural model. Table 7 identifies the path coefficients (Coeff.) and p-values for the research model. In addition, we analyzed the hypothesized relationships using p-values obtained. As a result of the test for H1, it was found that gender has a significant effect on the perceived maliciousness of online news comments (coefficient = 0.092, *p*-value <0.01). In other words, women are more sensitive to the malicious comments than men. We also found that younger people are more sensitive to perceived maliciousness (coefficient= −0.065, *p*-value<0.05). Regarding H2, people with high empathy are perceived malicious comments more sensitively (coefficient = 0.176, *p*-value <0.01), but it was confirmed that morality does not affect. The more people think the comments are important, the less sensitive they are to malicious comments (coefficient = −0.093, *p*-value <0.05), supporting H3. Regarding H4, the people read more comments, the more sensitive they are to malicious comments (coefficient = 0.170, *p*-value <0.01), supporting H4. It is found that the involvement of the news issue does not impact the perceived maliciousness, rejecting H5. As a result of the test for H6 and H7, the more people believed that other people wrote malicious comments, the less sensitive they were to malicious comments (coefficient = −0.140, *p*-value <0.01), but the ratio of malicious comments in the article does not affect the perceived maliciousness in online news comments.

**Table 7:** Results of hypotheses tests.

Hypothesis	Coeff.	Hypothesis Supported
H1-1: Gender → Perceived maliciousness	0.092***	Supported
H1-2: Age → Perceived maliciousness	−0.065**	Supported
H2-1: Morality → Perceived maliciousness	0.037	Not Supported
H2-2: Empathy → Perceived maliciousness	0.176***	Supported
H3: Attitude → Perceived maliciousness	−0.093**	Supported
H4: Reading Amount→ Perceived maliciousness	0.170***	Supported
H5: Issue involvement → Perceived maliciousness	−0.040	Not Supported
H6: Presence of malicious comments → Perceived maliciousness	−0.005	Not Supported
H7: Perceived peer’ behavior on malicious commenting → Perceived maliciousness	−0.140***	Supported
*R* ^2^	0.136	
Obs	1000	

Dependent variable: perceived maliciousness. **p < 0.05; ***p < 0.01.

In addition, according to the survey data in the study, only 17 out of 1000 respondents answered that they have experience of writing malicious comments. The 17 people have a significantly lower perceived maliciousness of online news comments compared to the people who have no experience of writing malicious comments (*t* value: −3.57, *p* <0.01, malicious comment writing group: Perceived Maliciousness *M* = 5.08, SE = 0.39, non-malicious comment writing group: Perceived Maliciousness *M* = 6.00, SE = 0.03). In other words, the low the perceived maliciousness of online comment is, the higher the likelihood of writing malicious comments.

## 4. Discussion and conclusion

This study tried to examine a comprehensive model for the perceived maliciousness of online news comments. The findings are summarized as follows. For the impacts of individual factors on the perceived maliciousness of online comment, we examine the demographic factors (e.g. gender and age) and personalities (e.g. morality, and empathy), attitude and reading amount. The results indicate that females are found to have high in perceived maliciousness of online news comments. In addition, age can affect the perceived maliciousness of comment, where the older has low perceived maliciousness in online news comment. It was found that empathy is positively influences to perceived maliciousness. Empathy is the ability to understand other people's situations, and it is interpreted as a sensitive reaction to malicious comments as the empathy for the target person of the malicious comment increases. On the other hand, it was expected that people with high morality would have perceived maliciousness, but they were not. Basically, moral reasoning process strongly depends on the culture or environment (Gibbs, 1992), therefore, the lack of previously developed policies lead to less guilt for new type of cyber deviant behavior (Kshetri, 2009). Accordingly, there are possibility that morality in readers’ mind did not preciously developed for cybercrime. In addition, about the relationship between attitude toward comments and perceived maliciousness, even though there is a problem with comments, those who have positive attitudes toward comments were found to be fewer perceived maliciousness of online news comments. In addition, it turns out that people who read a lot of comments regularly are more precise perceived maliciousness of online news comments. It was expected that if you read a lot of comments, you would be insensitive to the maliciousness in online news comments, but rather, the more they read comments, the more perceived maliciousness to online news comments. There is one possible interpretation that people who read a lot of comments might read helpful and productive comments and gain more utility of online news comments than who rarely read the news comments. Therefore, to specifically understand the impacts of reading amount on perceived malicious news comments, it is better to measure the amount of malicious comment that people read recent duration to control the impacts of frequency of malicious comments exposure.

For the impacts of contextual factors on the perceived maliciousness of online comment, we examine the impact of issue involvement, the presence of malicious comments in the same news article, and perceived peer’ behavior on malicious commenting. Unlike our expectation, issue involvement does not influence to perceived maliciousness to online news comments readers. This provided scenario in the current study pertains to an issue that aligns with individuals' interests, such as sports, rather than being directly associated with personal gain. Therefore, individuals may form perceptions of malicious comments regardless of their involvement, as the issue is relevant to their interests. However, for future research, it is necessary to observe the role of issue involvement from a diversified perspective. This can be achieved by introducing issues that are directly related to personal gain, health, or risks, allowing us to examine how perceptions of malicious comments may vary based on the degree of involvement. In addition, to understand the influence of the other malicious comments as contextual factors, we provide two types of comments list to the survey respondent. The results indicate that the presence of malicious comments in the new articles’ comment section does not affect the perceived maliciousness of online news comments. According to previous research on online news comments and their impacts on the opinions toward news articles, the online news comments specifically provides others’ opinion and help to form an perceptions on certain issues (Lee and Jang, 2009). However, the perceived maliciousness much related to the moral attitude or norm related area not issue related perceptions which might relatively easy to form in individual’s mind. In addition, the results indicate that people perceived the more peers are writing malicious comments, the lower the perceived maliciousness of online news comments is.

There are several theoretical contributions in this research. First, we extend previous research on perceived maliciousness in online news comments. Previous studies on perceptions on malicious comments mainly focused on the individual factors such as demographic characteristics (Anderson et al., 2014; Bogolyubova et al., 2018) and psychological factors (Craker and March, 2016; Bogolyubova et al., 2018; Gylfason et al., 2021) and individual behavioral characteristics (Coe et al., 2014; Hmielowski et al., 2014). According to Kenski et al. (2020), there are limited previous studies on the perceived maliciousness of online news comments. Their study focused on individual factors such as personality and news consumption patterns affecting the perception of malicious comments. We extend the scope of individual characteristics to attitude toward online comment, and personality such as morality, and empathy. In addition, we include contextual factors such as issue involvements, perceived peer’ behavior on malicious commenting and the presence of other malicious comments on the same news article. To the best of our knowledge, no existing study has validated the impacts of such contextual factors on the perceived maliciousness of online news comments. Second, this research tries to understand the possible bias about direct questions toward commenting behaviors results in investigating perceptions on maliciousness on online news comments. Compared to previous research, most of other researchers directly investigate the commenting behaviors or analyze the patterns of malicious comments (Coe et al., 2014; Bogolyubova et al., 2018; Gylfason et al., 2021). However, it is unreasonable to expect an honest answer to the question, “Are you writing malicious comments?”. For this reason, Jang et al. (2016) conducted to examine the factors of posting benevolent comments to indirectly derive the factors of posting malicious comments. To overcome the possible bias and understand the fundamental mechanisms of individual’s perceptions on deviant behavior, we composite the comprehensive model for perceived maliciousness for online news comments readers, not the writers. Third, existing malicious comments and comment-related studies have mainly focused on comments in the political field, where individual biases or opinions are obvious. However, this study controls the personal prior attitude on topics by focusing on articles in the field of sports.

The practical implications are as follows. Before discussing ways to minimize malicious comments, it is necessary to establish a social standard for which comments are defined as malicious comments. However, there is still ambiguity in the concept of malicious comments. From the standpoint of platform operators, in order to quickly delete and block malicious comments, it is necessary to prepare standards for which comments should be defined as malicious comments. In other words, to reduce the problem of malicious comments effectively, it is necessary to prepare a social consensus on the perceptions of malicious comments. However, there are lack of agreed definitions and perceptions of maliciousness in online news comments motivate our research. In this study, by examining in detail how the individual's perceived maliciousness of online news comments varies, it will be helpful to establish a criterion for malicious comments in consideration of users' perceptions, which will help in establishing an effective policy. To recognize the problem of malicious comments seriously is one of the ways to voluntarily reduce malicious comments. Therefore, digital literacy education can be conducted using the findings of the study as a guideline for the perceived maliciousness of online news comments. Based on the finding of the study, different approaches for malicious comment literacy may be applied depending on gender. We need to know that malicious comment literacy education centered on empathy rather than morality is necessary. In addition, it is necessary to emphasize that a very small number of people, not the general public, are writing malicious comments as a way to reduce the writing of malicious comments. Additionally, perceived maliciousness of comments can be influenced by their own or others' attitudes toward such comments. Therefore, it is important to continuously raise awareness of the severity of the cyberbullying and the potential harm caused by malicious comments through education and campaigns, in order to promote appropriate attitudes toward them. Ultimately, researching the factors that affect users' perception of malicious comments can help reduce their prevalence and create a healthier and safer online communication environment for all.

This study contents the importance of individuals’ perceived maliciousness of online news comments. For future research, this research can be a bridge of the behavior of writing malicious comments and various antecedents. To consider the relationship between the perceived maliciousness in online comment and the behavior of writing malicious comments from the survey data, this study would suggest future research about the moderating and mediating factors that affect the perceived maliciousness of online comment and writing malicious comments. In the case of elder abuse, researchers investigate the perception of abusive behavior before revealing the causes of abuse to prevent it (Melchiorre et al., 2014). In this context, the perpetrator is likely to commit a crime due to the perpetrator's cognitive distortion. Therefore, the perpetrator is treated by reeducating the perceptions toward illegal behavior (Aos et al., 2001). Jung (2010) also contends that the intention of posting malicious comments is high in groups that perceive malicious comments as general norms. These existing studies can be used as evidence that perception of a particular behavior can increase the likelihood of action. Accordingly, the perceived maliciousness of online news comments can affect the writing of malicious comments. Therefore, revealing moderating or mediating factors that affect relationship between the perceived maliciousness of online news comments and writing behaviors can provide more systematic access to ways to improve the perception and, in conclusion, reduce the likelihood of writing malicious comments.

The limitations of this study and future studies are as follows. The limitation of this study is that it only examined the variables of individuals' demographic characteristics and context that could impact perceived maliciousness, without considering psychological factors or prior experiences that may also influence one's ability to recognize malicious comments and their level of understanding of the article. To address the limitations of this study, additional future research is needed to explore other factors that could impact perceived maliciousness. Second, it is necessary to conduct additional research on other countries to see if the perception of malicious comments is caused by cultural differences. Third, considering that this research deal with deviant behaviors of online platform, it is necessary to control the social desirability bias of respondents. For future research, the survey will include the social desirability bias to reduce the inherent bias from respondents. In addition, because we conduct survey in one time as cross-sectional format, although we examine the problems of common method bias as statistically, there are possible common method bias in cross-sectional forma survey. To solve the problems, the future research can develop the two-time survey by separating exploratory variables and dependent variables each. The limitations of this study are also as follows. In order to ensure an equal distribution of age and gender among participants, we set the proportions of males and females to 50% each, while including 20% of participants from each age group ranging from their 20s to their 60s. While it is characteristic of Korean society to have a high college enrollment rate, variations in educational levels could potentially influence the perception of malicious comments. Therefore, future research should consider designing studies that equalize educational backgrounds and include participants from the teenage demographic, as this would provide a more comprehensive understanding of the topic. Finally, in the present study, overall morality of individuals was measured and used as an independent variable. However, when it comes to issues like cybercrime where the boundaries are not clearly defined in society, individuals' perceptions and morality may not have been formed proactively. Therefore, future research should focus on proactively measuring the extent to which individuals have established a moral framework regarding malicious comments they generate or the societal issues resulting from malicious comments, especially when the ethical boundaries are not clearly defined.

## Data availability statement

The raw data supporting the conclusions of this article will be made available by the authors, without undue reservation.

## Ethics statement

Ethical review and approval was not required for the study on human participants in accordance with the local legislation and institutional requirements. The patients/participants provided their written informed consent to participate in this study.

## Author contributions

SL contributed to the review of perceived maliciousness in online environment. HB contributed measuring conceptual items. SL and HB contributed with the introduction, data collection, and analysis section. SK have reviewed and contributed to the final version of the text. HB and SK contributed with the original idea. All authors have written collaboratively the first version of the text, contributed to the development of the general logic of the main argument, article, and approved the submitted version.

## Funding

This work was supported by the Ministry of Education of the Republic of Korea, the National Research Foundation of Korea (NRF-2019S1A3A2099973), and the MSIT (Ministry of Science and ICT) Korea, under the ITRC (Information Technology Research Center) support program (IITP-2023-2020-0-01749) supervised by the IITP (Institute of Information & Communications Technology Planning & Evaluation).

## Conflict of interest

The authors declare that the research was conducted in the absence of any commercial or financial relationships that could be construed as a potential conflict of interest.

## Publisher’s note

All claims expressed in this article are solely those of the authors and do not necessarily represent those of their affiliated organizations, or those of the publisher, the editors and the reviewers. Any product that may be evaluated in this article, or claim that may be made by its manufacturer, is not guaranteed or endorsed by the publisher.

## Appendix A

“Choi Dong-soo, the Missed Bat: Need for Caution Even When Unintentional"

[By Lee Saerom, Reporter] On the afternoon of the 9th, the Korea League match between Team A and Team B took place in Seoul in 2020. In the bottom of the 2nd inning, Choi Dong-soo, the leadoff batter for Team B, swung and missed, letting go of his bat. The bat flew toward the A team's shortstop and landed on the ground, surprising everyone. Startled, Choi Dong-soo raised both hands in a gesture of apology toward the A team players, repeating the gesture several times.

Choi Dong-soo's actions did not appear intentional. Instead, his expression showed self-blame. However, not only in this game but also in his swing, Choi Dong-soo had some issues. When he swung and missed, his foot tended to go back too far, resulting in a higher bat angle. Even slight twisting of the body during Choi Dong-soo's swing could lead to dangerous moments. Correcting one's hitting mechanics, honed since childhood, is not easy. Nevertheless, for the sake of oneself and other players, it seems that more caution is needed when standing at the plate.

News Comment

1. Why on earth is this guy playing baseball? He's caused injuries to several people, yet he shamelessly continues. It's outrageous, to say the least. Now he's even acting all cheerful and carefree.
2. Oh, how typical… Always looking out for oneself, huh? If we want to put it nicely, it's just their way of expression… Holding the bat tightly because they can't achieve the desired swing, sticking to a form that can harm others, it may be commendable in a way, but causing harm or instilling fear in others… It's time to stop. It's really unpleasant to see how they intimidate players on the opposing team… How many players are there who hold onto the bat, refusing to let go, and send it toward the pitcher after a swing, with no intention of backing down? Well, I suppose it's all about the theoretical hitting technique…"
3. He's truly a piece of trash.
4. He's got some damn character issues, doesn't he?
5. Can't fix it? Bullshit. He's performing well, so he doesn't bother to fix anything. Ha! Even if opposing team catchers are bleeding, this guy doesn't give a damn. I'll be damn careful when he prances around the field. Just by looking at the comment section, it's obvious that he's a worthless player in so many ways. The era where being good at baseball forgives everything is long gone.
6. People can make mistakes too… Hang in there!
7. If he makes a mistake, they're always criticizing the player, so sad… Poor thing.
8. Not just once, but for the fucking nth time, it feels like he's tossing away a soldier's gun. Among hundreds of baseball players, I've never seen such a damn bastard.
9. He's like a fucking psychopath… Seems like he's scheming to take down anyone he doesn't like, trying to mess with their heads.
10. This fucker is just trash. He's trying to turn the atmosphere around and almost throws a tantrum, but the umpire doesn't even pay attention. Tsk tsk tsk.
